# Papillary Muscle Delayed Hyperenhancement: Prevalence and Clinical Implications in a Large Population With Dilated Cardiomyopathy

**DOI:** 10.1016/j.jacadv.2024.101103

**Published:** 2024-07-13

**Authors:** Casper W.H. Beijnink, Anne G. Raafs, Jacqueline L. Vos, Job A.J. Verdonschot, Maurits A. Sikking, Laura Rodwell, Stephane R.B. Heymans, Robin Nijveldt

**Affiliations:** a Department of Cardiology, Radboud University Medical Center, Nijmegen, the Netherlands; b Department of Cardiology, Cardiovascular Research Institute (CARIM), Maastricht University Medical Center, Maastricht, the Netherlands; c Department of Cardiovascular Research, University of Leuven, Leuven, Belgium; d Netherlands Heart Institute, Utrecht, the Netherlands

**Keywords:** cardiac magnetic resonance imaging, dilated cardiomyopathy, late gadolinium enhancement, prognosis

## Abstract

**Background:**

Papillary muscle–delayed hyperenhancement (papHE) at cardiac magnetic resonance indicates fibrotic or infiltrative processes. Contrary to myocardial HE, the prevalence and prognostic implications of papHE in patients with nonischemic dilated cardiomyopathy are unclear.

**Objectives:**

The purpose of this study was to determine the prevalence of papHE and describe its association with adverse clinical outcomes.

**Methods:**

This prospective cohort study included 528 patients who underwent late gadolinium enhancement cardiac magnetic resonance. The primary outcomes were all-cause mortality, sudden cardiac death, life-threatening arrhythmia, and hospitalization for heart failure. Patients were allocated into 4 categories: the first without papHE and without myocardial HE, the second with papHE, the third with myocardial HE, and the fourth with papHE and myocardial HE. The hazards of the primary outcomes for each category were compared using multivariable Cox regression.

**Results:**

papHE was present in 131 patients (25%). The median follow-up duration was 6.1 years (IQR: 3.7-9.7 years). Isolated papHE and isolated myocardial HE were not significantly associated with any of the prespecified outcomes. Patients who had both myocardial HE and papHE were at an increased risk of all-cause mortality (HR: 2.33, 95% CI: 1.26-4.30), sudden cardiac death (HR: 3.77, 95% CI: 1.59-8.94), life-threatening arrhythmia (HR: 3.94, 95% CI: 1.34-11.58), and hospitalization for heart failure (HR: 2.97, 95% CI: 1.30-6.80).

**Conclusions:**

The combined presence of myocardial and papHE was independently associated with adverse outcomes. Future studies should investigate if the incorporation of papHE and myocardial HE may improve clinical decision-making strategies to select dilated cardiomyopathy patients who would benefit the most from ICD implantation.

Nonischemic dilated cardiomyopathy (DCM) is defined as left ventricle (LV) dilation and systolic dysfunction, in the absence of significant coronary artery disease or pathological loading conditions such as valvulopathies, congenital heart disease, or hypertension.^1^ Patients with DCM are at an increased risk of cardiovascular mortality (6.6% percent at 3.8 years), heart failure hospitalizations (HFH), and ventricular arrhythmias.^2^ Implantable cardioverter-defibrillators (ICD) can be used to mitigate the risk of ventricular arrhythmias and consequent sudden cardiac death (SCD).^3^ However, the rate of SCD, and therefore the benefit from ICD implantation, is generally lower in DCM than in ischemic cardiomyopathy.^4^, ^5^, ^6^ Secondly, ICD implantation in DCM patients for primary prevention does not significantly reduce all-cause mortality.^6^ Therefore, additional selection criteria for ICD implantation are needed to select patients who would benefit the most from ICD implantation,^3^ in an effort to further reduce SCD rates without unnecessary treatment. One of the proposed criteria is the presence of subendocardial, midwall, and epicardial delayed hyperenhancement (HE) on late gadolinium enhancement (LGE) cardiac magnetic resonance imaging (CMR), which greatly magnifies the risk of (sudden) cardiac death, appropriate ICD shock, and HFH.^5^^,^^7^ Apart from myocardial HE, delayed hyperenhancement of the papillary muscle (papHE) is another promising factor that has recently been linked to ventricular arrhythmias and SCD in patients with ischemic cardiomyopathies, independently from the extent of myocardial HE.^8^ The exact prevalence and prognostic implications of papHE in DCM patients were unknown however. Therefore, this study aimed to describe the prevalence of papHE in DCM patients and to assess its incremental prognostic impact over myocardial HE. We hypothesized that papHE occurs in DCM patients and that its presence may be associated with increased mortality. If true, the presence of papHE in DCM may serve as a novel selection criterion for ICD allocation.

## Methods

For this retrospective analysis of a prospectively included cohort, consecutive patients with nonischemic DCM were included from the Maastricht Cardiomyopathy registry (NCT04976348), who were enrolled between 2003 and 2018. The exact selection criteria^9^ and other results^10^ have previously been published. Briefly, we included patients with DCM or hypokinetic non-DCM as defined according to international definitions,^11^ who are gathered under the term DCM. Both were defined as having an LV ejection fraction <50%. A diagnosis of DCM was made when the LV end-diastolic diameter >33 mm/m^2^ in men or >32 mm/m^2^ in women. Patients were diagnosed with hypokinetic non-DCM when the LV end-diastolic diameter ≤33 mm/m^2^ in men or ≤32 mm/m^2^ in women. The exclusion criteria for the registry were as follows: 1) the presence of significant coronary artery disease, defined as an epicardial stenosis >50% (as assessed by coronary artery angiography or computed tomography angiography); 2) prior myocardial infarction on LGE CMR; 3) primary valvulopathies; 4) the presence of congenital heart disease; 5) acute myocarditis; 6) arrhythmogenic cardiomyopathy; 7) hypertensive or hypertrophic cardiomyopathy and restrictive or peripartum cardiomyopathy; and 8) the presence of myocardial storage diseases. Of the 551 patients with available scans, LGE CMR images were missing in 4 cases, and LGE CMR images were of an uninterpretable quality in 19 cases. These patients were excluded from the analysis, resulting in a study population of 528 patients. The study was approved by the local ethics committee and performed in accordance with the Declaration of Helsinki. All patients gave written informed consent.

### Clinical follow-up and outcomes

All patients participated in elaborate outpatient work-up, including a physical exam and anamnesis. Clinical events were collected from the medical records and telephone contact with the general practitioner. Mortality data were extracted from the municipal registry. Starting from index CMR, the primary outcomes were recorded. These included: 1) all-cause mortality; 2) SCD, defined as unexpected mortality in a previously stable patient; 3) life-threatening arrhythmias (LTA), defined as nonfatal ventricular fibrillation and/or ventricular tachycardia with hemodynamic instability, with or without appropriate ICD shock; and 4) HFH, defined as admission for >24 hours with a primary diagnosis of heart failure or progressive deterioration of heart failure demanding intensified treatment.^12^

### CMR imaging

The CMR images were acquired on a clinical 1.5-T MRI scanner (Intera, Philips Medical Systems). The scanning protocol consisted of standard long axis 2-, 3-, and 4-chamber cine imaging. A short axis cine stack was acquired consequently, covering the entire LV. The cine images were acquired during end-expiratory breath holds, using a balanced steady-state free precession sequence of (repetition time: 3.0-3.5 milliseconds; echo time: 1.5-1.8 milliseconds: flip angle: 60°; temporal resolution: <50 milliseconds). Gadolinium-based contrast agents were administered at a dosage of 0.10 to 0.20 mmol/kg, followed by LGE CMR imaging 10 to 15 minutes post-contrast administration, using a 2-dimensional–segmented inversion-recovery–prepared gradient echo sequence and single shot LGE images, in the same orientation as the cine images. Contiguous LGE CMR stacks were acquired, covering the LV in the short axis and different long axis orientations. As such, the papillary muscles could be assessed from multiple angles over their full trajectory.

### CMR image analysis

All analyses were performed with Medis (Medis Medical Imaging BV, version 2.0.48.8). From the short axis cine images, LV volumes and the LV end-diastolic and LV end-systolic volume were measured by manual contour tracing. Left atrial dimensions and left atrial reservoir strain were computed following a previously published protocol.^10^ The LV ejection fraction was calculated as: (LV end-diastolic volume−LV end-systolic volume)/LV end-diastolic volume × 100. Myocardial HE was measured semiquantitatively using the full-width at half maximum technique^13^ and expressed as a percentage of end-diastolic LV mass.

**Figure 1:**
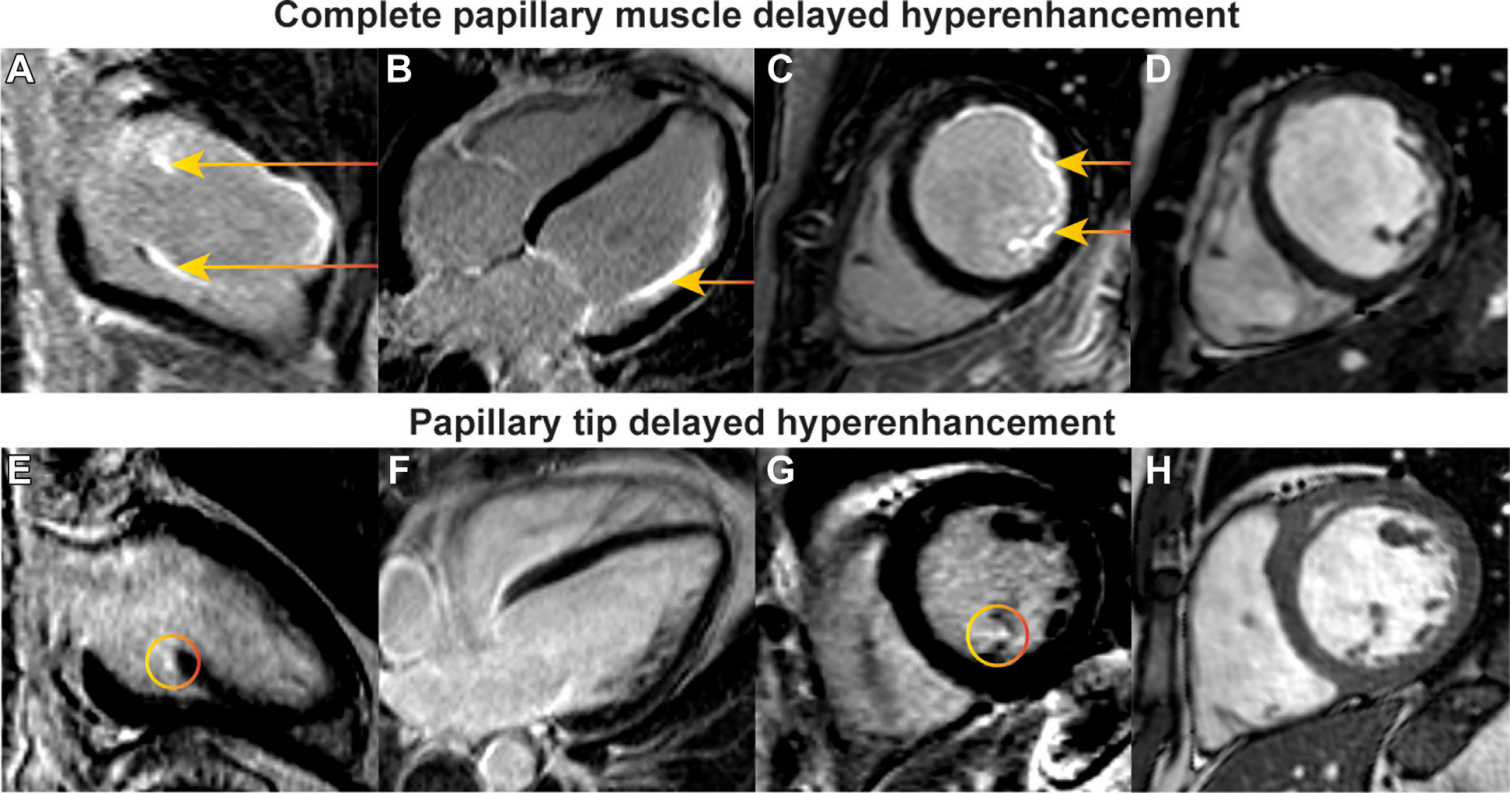
Delayed Hyperenhancement of the Papillary Muscle and Papillary Muscle Tip Delayed hyperenhancement of the papillary muscle vs the papillary tip (A to D) are respectively 2-chamber LGE CMR images, 4-chamber LGE CMR images, short-axis LGE CMR images, and short-axis end-diastolic cine frames of a patient with marked papHE. (E to H) The same frames of a patient with HE of the papillary tip only. The arrows mark papHE and the circles papillary tipHE. CMR = contrast-enhanced cardiac magnetic resonance imaging; HE = delayed hyperenhancement; LGE papHE = papillary muscle delayed hyperenhancement.

The presence of papHE was assessed visually and blinded from patient outcomes, following 3 steps. First, an experienced investigator identified the supero-lateral and infero-medial papillary muscle bundles on short axis cine images, which may consist of several muscle heads. The number of heads per group was counted as demonstrated in Supplemental Figure 1. Consequently, the papillary muscles were identified on the LGE CMR images at the same slice position. The presence of papHE was defined as HE in a papillary muscle head in 2 contiguous LGE CMR slices and its presence needed to be confirmed on the long-axis LGE CMR slices. If the 2D inversion recovery gradient echo LGE CMR images were of insufficient quality, papHE was evaluated on the single shot images. Difficult cases were discussed with a senior CMR physician with >15 years of experience, until consensus was reached. If papHE was seen in only 1 basal slice adjacent to the chordae tendineae and confirmed in a long-axis LGE CMR view, this was defined as papillary tip HE (tipHE). Figure 1 contains an example of complete papHE and tipHE. Consequently, the nature and extent of papHE were specified according to location, completeness, and the number of slices with papHE. The location could be superolateral, inferomedial, or double papHE. PapHE was called partial, if the extent was ≤50% of the papillary muscle head or complete if the extent exceeded 50%. To semiquantitively measure the extent of papHE, we used a score in which a muscle head with complete papHE counted for 2 points, partial HE for 1 point, and absence of HE for 0 points. The total of points was divided by the number of papillary muscle heads and multiplied by the ratio between slices that contained papHE and the total number of slices on which the papillary muscles were visible.

### Statistical methods

Continuous variables were expressed as mean ± SD or as median (IQR) in case of non-normal distribution and compared using the Student T test and Mann Whitney U test as appropriate. Dichotomous variables are presented as a number with percentage and compared using the Pearson chi-square test. To assess the incremental prognostic value of papHE over myocardial HE, patients were allocated into 4 groups, the first without myocardial HE or papHE, the second with papHE only, the third with myocardial HE, and the fourth with papHE and myocardial HE. The median follow-up time was based on the time that elapsed between enrollment in the registry and the date of last follow-up or mortality and was calculated using the inverse Kaplan-Meier method. For each of the (composite) events of interest, the exact time to event was calculated as the elapsed time between the date of enrollment in the registry and the date on which the relevant event occurred. Patients who had not experienced the event of interest were censored at data cut-off. Cumulative incidence functions were plotted which were tested for significance using Gray’s test. HRs were computed using univariable Cox regression for different parameters of known prognostic importance. Consequently, multivariable models were constructed to investigate the hazard of papHE with or without myocardial HE, with regard to the main outcomes. The hazard ratios for the presence of papillary HE, myocardial HE, and papillary and myocardial HE were computed with neither papillary nor myocardial HE as the reference group. Correction was applied for age (continuous), sex (males reference), and LV ejection fraction (continuous). The presence of tipHE was not considered in the multivariable models, as its prognostic impact was expected to be weak and colinear with that of papHE. To account for competing risks, a Fine-Gray subdistribution hazard model was fitted, correcting for non-sudden cardiac death in the case of SCD. In case of nonfatal outcomes, all-cause mortality was considered a competing risk.^14^ To prevent overfitting of the statistical models, a separate model was run that corrected for LVEF only.

As a secondary objective, to assess the association between the extent of papHE and patient outcomes, patients were allocated into 4 groups according to the extent of papHE, the first being those without papHE and without tipHE, the second group with tipHE but without papHE, the third with papHE score ≤ median, and the fourth group with papHE score > median. Group 2, 3, and 4 were sequentially compared to those without papillary tipHE and without papHE.

## Results

### Patient characteristics

The baseline characteristics are summarized in Table 1. Of 528 patients, 131 (25%) had papHE. The location of papHE was exclusively superolateral in 15 cases (3%) and inferomedial in 45 cases (8%). Seventy-one patients (13%) had papHE in both papillary muscles. Patients who had papHE were much more likely to have tipHE as well (130 [99%] of patients with papHE had tipHE, as compared to 195 [49%] patients without papHE, *P* < 0.001). Patients with papHE were older (58 ± 12 years vs 52 ± 14 years, *P* < 0.001) and more likely to use diuretics (71 [57%] vs 152 [40%], *P* < 0.001). Patients with papHE had 642 papillary muscle heads in total (5 ± 1 head per patient) of which 299 (47%) showed papHE. The papHE was partial in 262 (88%) and complete in 37 (12%) of the contrast-enhanced muscle heads. Patients with papHE had a lower LV ejection fraction than those without (LV ejection fraction 32% ± 12% vs 36% ± 12%, *P* < 0.001), enlarged left atrial volumes, and impaired left atrial strain. Importantly, myocardial HE was more prevalent in those with papHE (91 [70%] vs 117 [30%], *P* < 0.001). See Table 2 for the baseline CMR characteristics.

**Table 1:** Patient Characteristics

	Total (N = 528)	papHE (n = 131)	No papHE (n = 397)	*P* Value
Demographics				
Age, y	54 ± 13	58 ± 12	52 ± 14	<0.001
Male	337 (64)	90 (69)	247 (62)	0.180
DCM	346 (66)	91 (70)	255 (64)	0.274
HDNC	182 (35)	40 (31)	142 (36)	0.274
Cardiovascular risk factors				
Chronic obstructive pulmonary disease	45 (9)	13 (10)	32 (8)	0.508
Hypertension	156 (30)	42 (32)	114 (29)	0.467
Dyslipidemia	79 (15)	15 (12)	64 (16)	0.194
Diabetes mellitus	57 (11)	9 (7)	48 (12)	0.095
Positive family history	92 (17)	25 (19)	67 (17)	0.564
Atrial fibrillation	94 (18)	29 (22)	65 (16)	0.135
Post-discharge medication				
Beta blocker	372 (73)	97 (78)	275 (71)	0.138
ACE inhibitor, ARB, ARNI	409 (80)	102 (82)	307 (80)	0.539
MRA	153 (30)	38 (31)	115 (30)	0.870
Diuretics	223 (44)	71 (57)	152 (40)	<0.001
NYHA functional class				
I	257 (49)	56 (43)	201 (51)	0.054
II	199 (38)	52 (40)	147 (37)	
III	65 (12)	23 (18)	42 (11)	
IV	7 (1)	0 (0)	7 (2)	

Values are mean ± SD or n (%).

ACE = angiotensin-converting enzyme; ARB = angiotensin II receptor blocker; ARNI = angiotensine receptor neprilysin inhibitor; DCM = dilated cardiomyopathy; HNDC = hypokinetic nondilated cardiomyopathy; MRA = mineralocorticoid receptor antagonist; papHE = papillary muscle delayed hyperenhancement.

**Table 2:** Baseline CMR Parameters

	Total population	papHE (n = 131)	No papHE (n = 397)	*P* Value
Indexed LV end-systolic volume, ml/m^2^	73 (56-108)	84 (62-114)	70 (53-101)	<0.001
Indexed LV end-diastolic volume, ml/m^2^	120 (98-149)	128 (107-158)	116 (93-147)	0.001
LV ejection fraction (%)	35 ± 12	32 ± 12	36 ± 12	<0.001
Indexed left atrial volume (mL/m^2^)	35 ± 23	41 ± 23	33 ± 22	<0.001
Left atrial reservoir strain (%)	24 ± 11	20 ± 10	26 ± 11	<0.001
HE size (% of LV)	3 (1-7)	4 (1-10)	2 (1-4)	<0.001
Any myocardial HE	208 (39)	91 (70)	117 (30)	<0.001
Mid- or subendocardial HE	198 (38)	88 (67)	110 (28)	<0.001
Epicardial HE	49 (9)	20 (15)	29 (7)	0.006
HE of the papillary tip	325 (62)	130 (99)	195 (49)	<0.001

Values are in median (IQR), mean ± SD, or n (%).

CMR = cardiac magnetic resonance imaging; HE = delayed hyperenhancement; LV = left ventricle; papHE = papillary muscle delayed hyperenhancement.

### Primary outcomes

**Figure 2:**
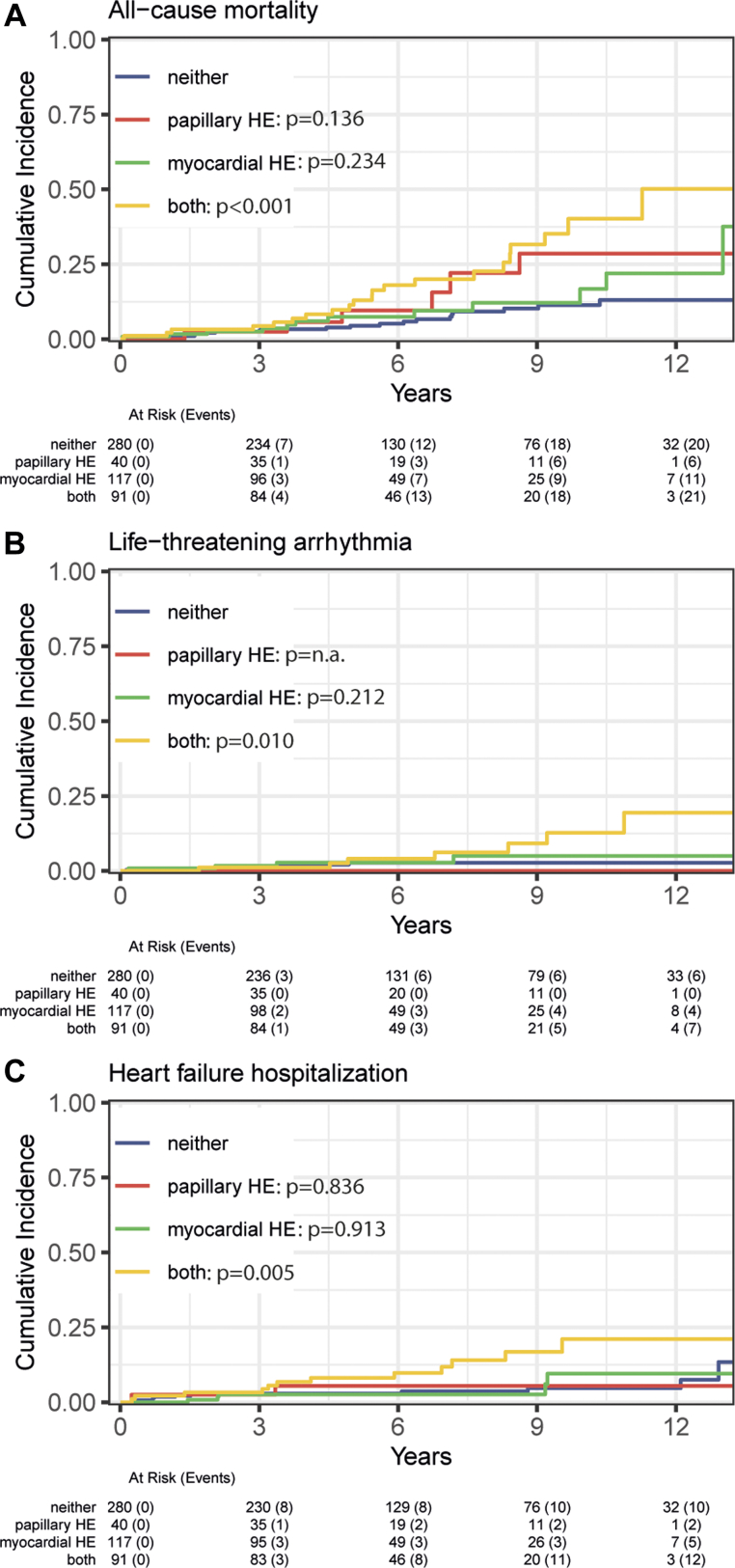
**Prognostic Implications of papHE and Myocardial HE on All-Cause Mortality, Life-Threatening Arrhythmias, and Hospitalization for Heart Failure** (A) Shows the cumulative incidence function for all-cause mortality, (B) for life-threatening arrhythmia, and (C) for hospitalization for heart failure, based on the presence of papillary muscle–delayed hyperenhancement, myocardial-delayed hyperenhancement, or both. papHE = papillary muscle delayed hyperenhancement.

Median follow-up time from enrollment in the registry until death or censoring was 6.1 years (IQR: 3.7-9.7 years). During this period, 38 patients with papHE (29%) had a total of 65 events, and 52 patients without papHE (13%) had 79 events, see Supplemental Table 1. The simultaneous occurrence of myocardial and papillary HE consistently led to the highest incidence of all-cause and sudden death, HFH, and LTA, see Table 3. In univariable Cox regression analysis, there was a significant association between simultaneous occurrence of papHE and myocardial HE and all tested outcomes (Table 4). No significant association was observed between isolated papHE and any of the tested outcomes. Similarly, isolated myocardial HE had a borderline association with SCD only (HR: 2.55, 95% CI: 0.98-6.63). See Figure 2 and the Central Illustration for the unadjusted cumulative incidence functions of the main outcomes. In multivariable analysis, the presence of isolated papHE and isolated myocardial HE were not associated with any of the main outcomes. In contrast, the simultaneous presence of myocardial HE and papHE was associated with all-cause mortality (HR: 2.33, 95% CI: 1.26-4.30), SCD (HR: 3.77, 95% CI: 1.59-8.94), LTA (HR: 3.94, 95% CI: 1.34-11.58), and HFH (HR: 2.97, 95% CI: 1.30-6.80), see Table 4. The effect sizes were similar in the reduced model that corrected for LVEF only. No difference in effect size was observed between the Cox multivariable models and the Fine-Gray subdistribution hazard models (see Supplemental Table 2).

**Table 3:** Clinical Event Rates

	No HE (n = 280)	papHE Only (n = 40)	Myocardial HE Only (n = 117)	Papillary and Myocardial HE (n = 91)	*P* Value
All-cause mortality	22 (8)	6 (15)	12 (10)	21 (23)	0.001
Sudden cardiac death	9 (3)	3 (8)	8 (7)	13 (14)	0.002
Heart failure hospitalization	12 (4)	2 (5)	5 (4)	12 (13)	0.014
Life threatening arrhythmia	6 (2)	0 (0)	5 (4)	8 (9)	0.015
Patient level event rate	32 (11)	7 (18)	20 (17)	31 (34)	0.001

Values are n (%).

HE = delayed hyperenhancement; papHE = papillary muscle delayed hyperenhancement.

**Table 4:** The Effect of Papillary and Myocardial Delayed Hyperenhancement on Clinical Outcomes

	Univariable HR	*P* Value	Multivariable HR	*P* Value
All-cause mortality				
Papillary HE	1.99 (0.80-4.90)	0.137	1.24 (0.49-3.11)	0.648
Myocardial HE	1.51 (0.75-3.07)	0.249	1.41 (0.69-2.87)	0.343
Papillary and myocardial HE	**3.17 (1.74-5.77)**	**<0.001**	**2.33 (1.26-4.30)**	**0.007**
Age	**1.04 (1.02-1.07)**	**<0.001**	**1.03 (1.01-1.06)**	**0.005**
Sex (female)	0.65 (0.37-1.14)	0.134	0.68 (0.38-1.19)	0.177
LV ejection fraction	**0.96 (0.94-0.98)**	**<0.001**	**0.97 (0.95-0.99)**	**0.005**
Sudden cardiac death				
Papillary HE	2.42 (0.65-8.95)	0.187	1.65 (0.44-6.27)	0.460
Myocardial HE	2.55 (0.98-6.63)	0.055	2.48 (0.94-6.50)	0.066
Papillary and myocardial HE	**4.91 (2.09-11.53)**	**<0.001**	**3.77 (1.59-8.94)**	**0.003**
Age	1.02 (0.99-1.04)	0.248	1.00 (0.97-1.03)	0.846
Sex (female)	0.45 (0.19-1.03)	0.059	0.47 (0.20-1.10)	0.082
LV ejection fraction	**0.95 (0.92-0.98)**	**<0.001**	**0.96 (0.93-0.99)**	**0.004**
Life-threatening arrhythmia				
Papillary HE	0.00 (0.00-xx)^a^	0.985	0.00 (0.00-xx)^a^	0.985
Myocardial HE	2.23 (0.68-7.30)	0.187	2.09 (0.63-6.93)	0.228
Papillary and myocardial HE	**4.26 (1.37-12.33)**	**0.007**	**3.94 (1.34-11.58)**	**0.013**
Age	0.99 (0.95-1.02)	0.390	0.98 (0.94-1.01)	0.148
Sex (female)	0.79 (0.30-2.07)	0.628	0.88 (0.33-2.37)	0.798
LV ejection fraction	**0.96 (0.93-1.00)**	**0.048**	**0.96 (0.93-1.00)**	**0.045**
Heart failure hospitalization				
Papillary HE	1.25 (0.28-5.58)	0.775	1.07 (0.23-4.94)	0.927
Myocardial HE	1.10 (0.39-3.12)	0.865	0.97 (0.34-2.79)	0.957
Papillary and myocardial HE	**3.36 (1.50-7.52)**	**0.003**	**2.97 (1.30-6.80)**	**0.010**
Age	1.01 (0.98-1.04)	0.516	1.00 (0.97-1.03)	0.852
Sex (female)	1.23 (0.60-2.52)	0.566	1.41 (0.68-2.91)	0.353
LV ejection fraction	**0.96 (0.94-0.99)**	**0.011**	**0.97 (0.94-1.00)**	**0.026**

Values are HR (95% CI). Significant values are highlighted in **bold**. The hazard ratios for the presence of papillary HE, myocardial HE, and papillary and myocardial HE were computed with neither papillary or myocardial HE as the reference group. Correction was applied for age (continuous), sex (males reference), and LV ejection fraction (continuous).

HE = Delayed hyperenhancement; LV = Left ventricle; papHE = papillary muscle delayed hyperenhancement.

a No life-threatening arrhythmias were seen in patients with isolated papHE.

**Central Illustration:**
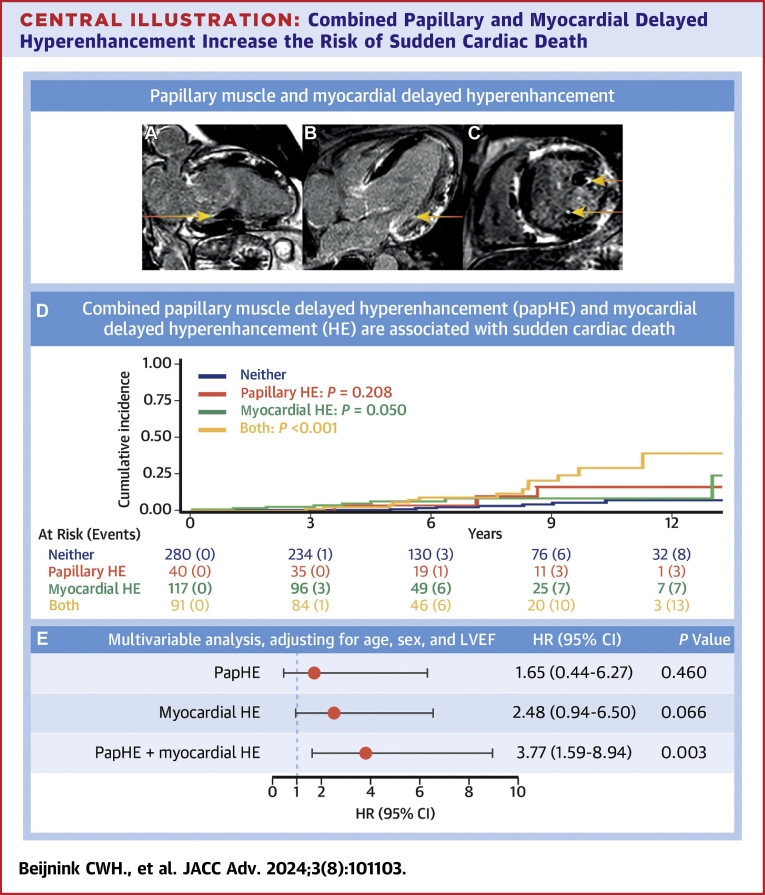
**Combined Papillary and Myocardial Delayed Hyperenhancement Increase the Risk of Sudden Cardiac Death** (A to C) 2-chamber, 4-chamber, and short axis LGE CMR images of a patient with papHE and myocardial HE. (D) Cumulative incidence function based on the presence of papillary HE, myocardial HE, or both, with regard to the incidence of sudden cardiac death. (E) Multivariable HRs of isolated and combined papHE and myocardial HE, after correction for age, sex, and LVEF. The hazard ratios for the presence of papillary HE, myocardial HE, and papillary and myocardial HE were computed with neither papillary or myocardial HE as the reference group. Correction was applied for age (continuous), sex (males reference), and LV ejection fraction (continuous). HE = delayed hyperenhancement; LGE CMR = contrast-enhanced cardiac magnetic resonance imaging; LVEF = left ventricular ejection fraction; papHE = papillary muscle delayed hyperenhancement.

Additionally, the effect of papHE increased with its extent. For instance, tipHE was not associated with any of the main outcomes, whereas papHE ≤ median score was associated with all-cause mortality and HFH. A papHE score above the median was significantly associated with all events except for LTA, with a higher HR than papHE scores ≤ median (see Supplemental Table 3).

## Discussion

We investigated the prevalence of delayed HE of the papillary muscles in patients with DCM and its incremental prognostic impact over myocardial HE. Two-thirds of the patients had tipHE and 1 in 4 patients had papHE. Although tipHE, isolated myocardial HE, and isolated papHE were not significantly associated with adverse outcomes, simultaneous papHE and myocardial HE increased the risk of all-cause mortality, SCD, LTA, and HFH, after correction for demographic and LV functional covariables.

### Prevalence of papillary muscle delayed hyperenhancement and clinical outcomes

In a referred population that was scanned for cardiomyopathy indications, papHE was found in 29 out of 149 patients (19%) with a nonischemic cardiomyopathy.^15^ There, the presence of papHE was not associated with all-cause mortality, worsening heart failure, and cardiac-related hospitalization after 3 years. We studied a larger population with extensive follow-up, assessing HFH and different types of mortality, as well as LTA. In line with earlier work, papHE was found in a quarter of DCM patients. On the other hand, in the presence of myocardial HE, papHE was associated with adverse outcomes. These associations were independent from demographic covariables and LV ejection fraction. The presence of isolated tipHE without involvement of the papillary muscle, however, was not associated with any of the studied outcomes. Additionally, combined myocardial and papHE led to a higher risk of SCD and LTA as compared to myocardial HE alone. In conclusion, papHE is seen in a quarter of DCM patients and its presence is associated with a broad spectrum of clinical adverse events, mainly when papHE coexists with myocardial HE.

### Pathophysiological mechanisms

An expansion of the extracellular matrix is a typical feature that unites ischemic^16^ and nonischemic cardiomyopathies.^17^ The extracellular matrix can have different microscopical phenotypes depending on the causal disease. Phenotypes may range from replacement fibrosis and sarcoid depositions to amyloid depositions. Gadolinium-based contrast agents wash out of the expanded extracellular matrix at a slower pace, due to relative hypoperfusion or an increased affinity for said environment, causing delayed HE.^18^ First, we demonstrate that papHE results in the increased rates of LTA and SCD on 1 hand and HFH on the other. Firstly, we speculate that papHE may lead to HFH as the papillary muscle contains contractile myocardial units^19^ and if these are lost, LV contraction may be impaired as a consequence. Indeed, patients with papHE have impaired LV ejection fraction and LA strain, implying reduced systolic and diastolic LV function. Second, previous studies found that papillary involvement in nonischemic cardiomyopathies may result in significant mitral valve regurgitation.^20^^,^^21^

Aside from HFH, papHE was associated with LTA and with SCD. In patients with ischemic cardiomyopathy and patients with mitral valve prolapse, the papillary muscle with delayed HE acts as a re-entry site for electric signals and gives rise to ventricular arrhythmias.^22^^,^^23^ In contrast, arrhythmogenicity of papillary muscles in nonischemic cardiomyopathies has not been assessed before. As we did not perform histologic assessment or electro-anatomical mapping, this mechanism remains speculative and should be confirmed in future experimental research.

### Study limitations

The main limitation was the limited sensitivity of conventional LGE CMR for papHE.^18^ In this study, gray blood LGE CMR was performed. It may be difficult to discern the papillary muscle with HE from the blood pool, especially as HE in DCM is often subtle and of a small quantity. This was combated by applying a strict protocol for CMR acquisition that included contiguous LV coverage in the short and long-axis LGE CMR views. Secondly, the short axis cine images were inspected simultaneously to minimize the chance of missing papHE. Finally, this registry enrolled participants in an outpatient setting. This population is well-treated and clinically relatively stable and low-risk, as can be deducted from the low clinical event rates. Our findings may therefore not apply equally to patients with more advanced disease who are not on intensive heart failure drug therapy.

### Clinical implications

Currently, papillary muscles are often ignored when reporting a CMR scan. Nevertheless, papHE was associated with mortality, LTAs, and heart failure. Future ICD studies in DCM patients may apply stratification based on the presence of papHE and myocardial HE, to investigate whether this would improve ICD recommendation guidelines. Such an improvement is needed as DCM patients are still at a considerable risk of SCD,^6^ and strong recommendations for primary prevention of SCD are lacking, even when the LV ejection fraction ≤35%.^3^

## Conclusions

PapHE was seen in a quarter of DCM patients at LGE CMR. The simultaneous occurrence of PapHE and myocardial HE increased the risk of adverse events, independently from demographic covariables. Whether the incorporation of papHE in addition to myocardial HE improves ICD recommendation guidelines needs to be confirmed in future studies.

Perspectives **COMPETENCY IN MEDICAL KNOWLEDGE:** PapHE is a relatively frequent finding in patients with DCM. Its presence is often subtle and easily overlooked. However, papHE is associated with worse outcomes and may become a standard part of a CMR report.

**TRANSLATIONAL OUTLOOK:** DCM patients with papHE and myocardial HE fall in a high-risk category for sudden cardiac death, life-threatening arrhythmias, and HFH. Future prospective studies are needed to investigate if the incorporation of papHE in decision-making strategies could improve ICD allocation.

## Funding support and author disclosure

The authors have reported that they have no relationships relevant to the contents of this paper to disclose.
