# Supplementary material for: Papillary Muscle Delayed Hyperenhancement: Prevalence and Clinical Implications in a Large Population With Dilated Cardiomyopathy
Source: JACC Adv. 2024 Jul 13;3(8):101103. doi: 10.1016/j.jacadv.2024.101103 (PMC11298879; doi:10.1016/j.jacadv.2024.101103)
Supplement: Supplementary Tables 1-3 and Figure 1 [file mmc1.docx]

**Supplemental Table 1** Clinical event rates for patients with and without papillary muscle delayed hyperenhancement

|  | **papHE (n=131)** | **No papHE (n=397)** | **p-value** |
| --- | --- | --- | --- |
| All-cause mortality, n (%) | 27 (21) | 34 (9) | <0.001 |
| Sudden cardiac death, n (%) | 16 (12) | 17 (4) | 0.001 |
| Heart failure hospitalization, n (%) | 14 (11) | 17 (4) | 0.007 |
| Life threatening arrhythmia, n (%) | 8 (6) | 11 (3) | 0.075 |
| Patient level event rate, n (%) | 38 (29) | 52 (13) | <0.001 |

Values are in n (%).

*Abbreviations: papHE: papillary muscle delayed hyperenhancement*

**Supplemental Table 2** Multivariable Fine-Gray subdistribution models for the primary outcomes

|  | **Sudden cardiac death** | |
| --- | --- | --- |
|  | **Multivariable HR** | **p-value** |
| **Papillary HE** | 1.69 (0.45-6.39) | 0.437 |
| **Myocardial HE** | 2.49 (0.93-6.67) | 0.069 |
| **Papillary and myocardial HE** | **3.72 (1.62-8.56)** | **0.002** |
| **Age** | 1.00 (0.97-1.03) | 0.870 |
| **Sex (female)** | 0.48 (0.20-1.17) | 0.106 |
| **LV ejection fraction** | **0.96 (0.93-0.98)** | **0.002** |
|  | **Life threatening arrhythmia** | |
|  | **Multivariable HR** | **p-value** |
| **Papillary HE** | n.a.* | n.a.* |
| **Myocardial HE** | 1.96 (0.57-6.73) | 0.282 |
| **Papillary and myocardial HE** | **3.82 (1.28-11.41)** | **0.016** |
| **Age** | 0.97 (0.94-1.01) | 0.101 |
| **Sex (female)** | 0.91 (0.34-2.47) | 0.860 |
| **LV ejection fraction** | 0.96 (0.93-1.00) | 0.053 |
|  | **Heart failure hospitalization** | |
|  | **Multivariable HR** | **p-value** |
| **Papillary HE** | 1.05 (0.22-5.06) | 0.952 |
| **Myocardial HE** | 0.94 (0.33-2.70) | 0.912 |
| **Papillary and myocardial HE** | **2.82 (1.25-6.39)** | **0.013** |
| **Age** | 0.99 (0.96-1.02) | 0.67 |
| **Sex (female)** | 1.47 (0.71-3.03) | 0.302 |
| **LV ejection fraction** | **0.97 (0.94-1.00)** | **0.035** |

Values are hazard ratios and 95% confidence intervals with corresponding p-values. Significant hazard ratios are highlighted in bold. (*) No life-threatening arrhythmias were observed in patients with isolated papHE. The hazard ratios for the presence of papillary HE, myocardial HE, and papillary and myocardial HE were computed with neither papillary or myocardial HE as the reference group. Correction was applied for age (continuous), sex (males reference), and LV ejection fraction (continuous).

*Abbreviations: LV: Left ventricle; papHE: papillary muscle delayed hyperenhancement, HE: Delayed hyperenhancement*

**Supplemental Table 3** Papillary tip and muscle hyperenhancement extent and clinical outcomes

| **PapHE Score** | **All-cause mortality** | | **Sudden cardiac death** | | **Life-threatening arrhythmia** | | **Heart failure hospitalization** | |
| --- | --- | --- | --- | --- | --- | --- | --- | --- |
|  | **HR** | **p-value** | **HR** | **p-value** | **HR** | **p-value** | **HR** | **p-value** |
| **Papillary tip hyperenhancement** | 1.33 (0.66-2.65) | 0.427 | 1.11 (0.42-2.91) | 0.841 | 0.72 (0.22-2.36) | 0.584 | 2.09 (0.73-5.94) | 0.169 |
| **Papillary muscle hyperenhancement ≤ median score** | 2.26 (1.01-5.06) | 0.047 | 2.21 (0.74-6.60) | 0.157 | 1.38 (0.35-5.53) | 0.648 | 4.01 (1.27-12.65) | 0.018 |
| **Papillary muscle hyperenhancement > median score** | 3.59 (1.72-7.50) | <0.001 | 4.12 (1.56-10.87) | 0.004 | 2.45 (0.74-8.06) | 0.142 | 4.30 (1.36-13.59) | 0.013 |

All displayed hazard ratios were obtained from univariable analysis. The hazard ratios for the presence of papillary tip hyperenhancement, papillary muscle hyperenhancement ≤ median score, and papillary muscle hyperenhancement > median score, were computed with neither papillary nor myocardial HE as the reference group.

*Abbreviations: papHE: papillary muscle delayed hyperenhancement*

**Supplemental figure 1 Papillary muscle anatomy**


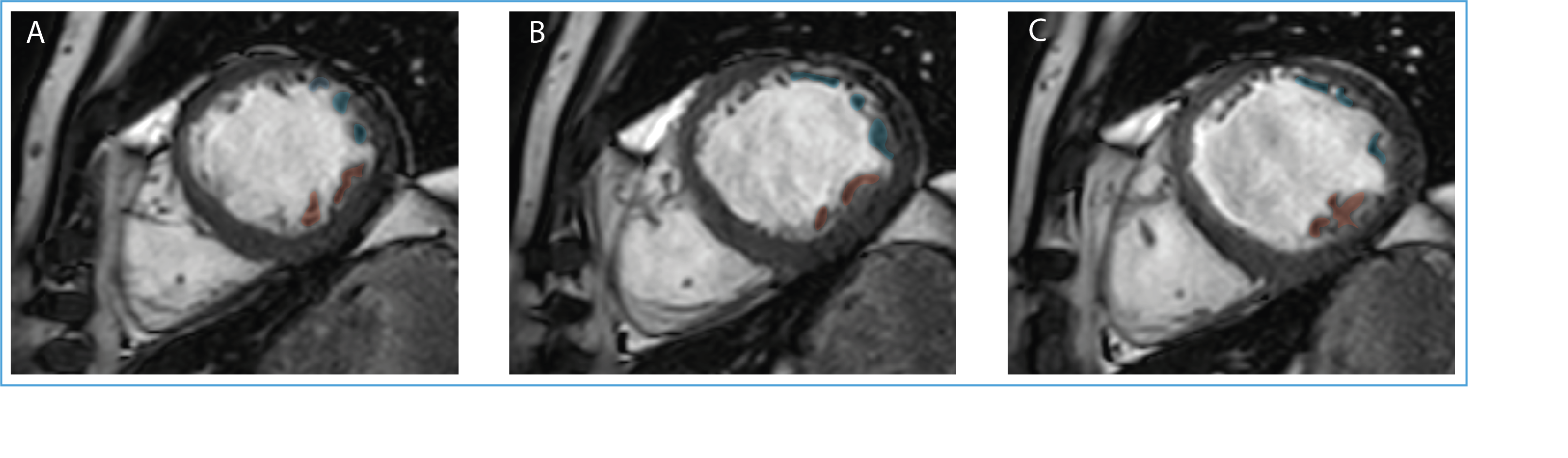


A-C show three contiguous short axis cine cardiac magnetic resonance imaging (CMR) slices. This patient has three superolateral papillary muscle bundles (blue) and two inferomedial papillary muscle bundles.
